# Acute chest pain in an elite rugby player: a case report of polyserositis with cardiac involvement

**DOI:** 10.1093/ehjcr/ytag784

**Published:** 2026-10-23

**Authors:** Theresa Maria Betz, Norbert Blank, Maura Magdalena Zylla, Birgit Friedmann-Bette

**Affiliations:** Department of Sports Medicine, Medical Clinic, University Hospital Heidelberg, Im Neuenheimer Feld 410, Heidelberg 69120, Germany; Department of Hematology, Onkology and Rheumatology, Medical Clinic, University Hospital Heidelberg, Im Neuenheimer Feld 410, Heidelberg 69120, Germany; Department of Cardiology, Angiology and Pneumonology, Medical Clinic, University Hospital Heidelberg, Im Neuenheimer Feld 410, Heidelberg 69120, Germany; Department of Sports Medicine, Medical Clinic, University Hospital Heidelberg, Im Neuenheimer Feld 410, Heidelberg 69120, Germany

**Keywords:** Polyserositis, Pericarditis, Athlete, Chest pain, C-reactive protein, Case report

## Abstract

**Background:**

The evaluation of chest pain is a common reason for athletes to seek consultation at a sports medicine clinic. Musculoskeletal causes are by far most frequent; however, rare and potentially life-threatening aetiologies must also be considered.

**Case summary:**

A 22-year-old professional rugby player presented with pleuritic chest pain for 2 weeks. Physical examination was unremarkable except for reduced breath sounds at the left lung base. Laboratory testing demonstrated marked systemic inflammation with a C-reactive protein level of 237 mg/L and mildly elevated high-sensitivity troponin T (21 pg/mL). Transthoracic ultrasound identified a fibrinous left-sided pleural effusion and a mild circumferential pericardial effusion. Subsequent cardiac magnetic resonance imaging (CMRI) confirmed the diagnosis of acute pericarditis. Comprehensive rheumatological evaluation established an autoimmune aetiology, consistent with a polyserositis secondary to mixed connective tissue disease (anti-nuclear antibody 1:80.000, speckled pattern, anti-RNP positive). Anti-inflammatory therapy with ibuprofen, colchicine and a corticosteroid pulse were initiated, resulting in clinical improvement and gradual normalization of laboratory and sonographic imaging findings within 6 weeks. After 3 months of sports restriction, follow-up CMRI demonstrated significant pericardial late gadolinium enhancement but without evidence of active inflammation. A gradual return-to-exercise protocol was subsequently implemented. Anti-inflammatory medication was discontinued after 6 months. The athlete has since remained clinically stable under regular follow-up for 1 year.

**Discussion:**

This case highlights the importance of thorough diagnostic evaluation of chest pain in young athletes, including consideration of autoimmune causes such as polyserositis. Despite structured follow-up, uncertainty persists regarding recurrence risk and its significance for long-term athletic participation.

Learning points

- The evaluation of chest pain in young athletes needs a thorough diagnostic to rule out serious conditions, even when they are rare.
- Young age and an autoimmune aetiology are factors associated with an increased risk of recurrence.
- The effect of exercise on recurrence risk and the development of constrictive pericarditis remains unclear.

## Introduction

Chest pain is a common reason for athletes to seek consultation at a sports medicine clinic. The causes of chest pain are diverse. In younger athletes (<35 years), musculoskeletal complaints or pulmonary causes are by far the most common aetiologies, whereas cardiac causes are very rare.^1^ Nevertheless, it is essential to rule out serious conditions, even if the symptoms appear innocuous.

## Summary figure

**Figure:**
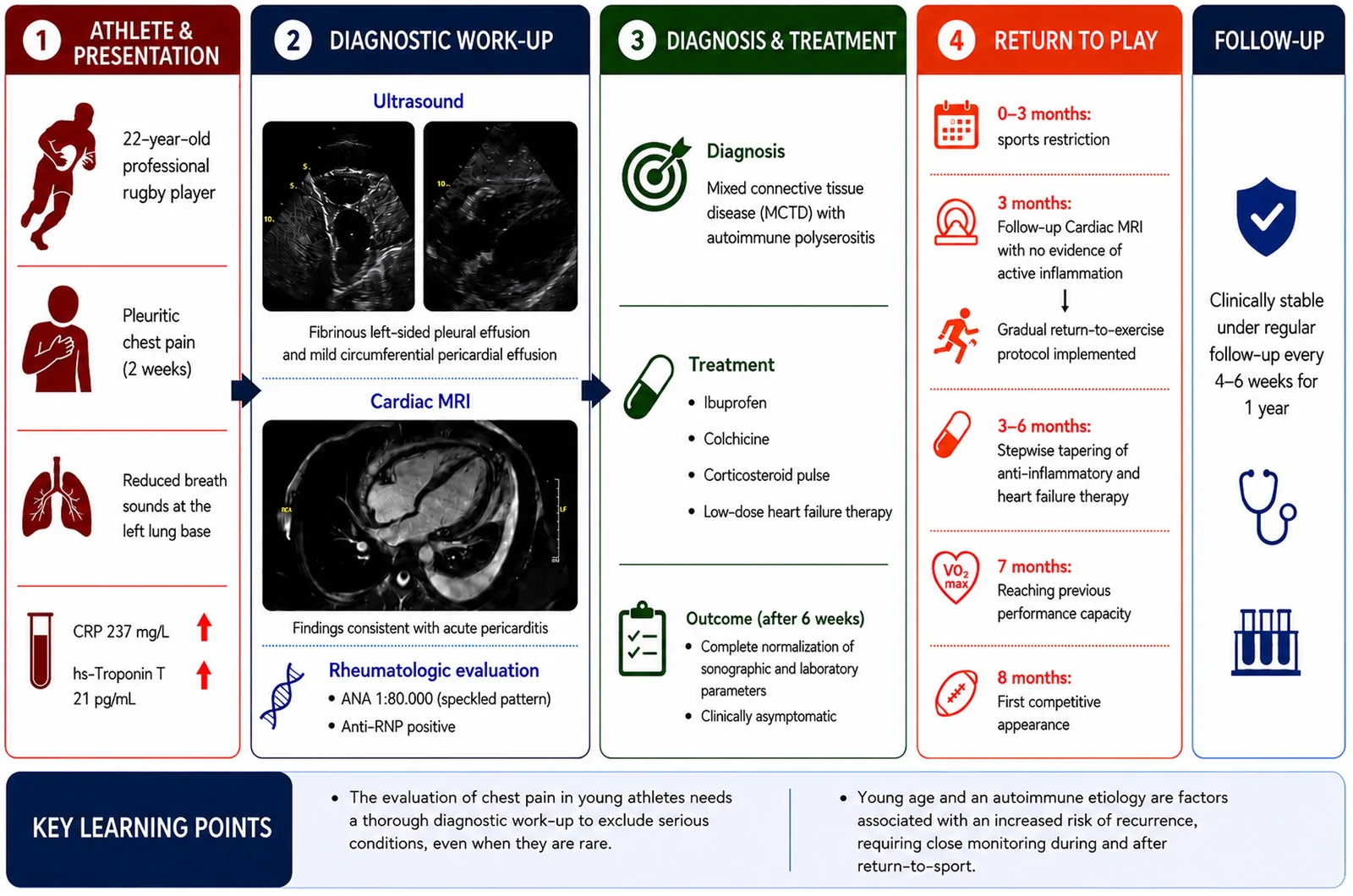


## Case presentation

We report the case of a 22-year-old professional rugby player with no prior internal medicine history who presented with pleuritic chest pain for 2 weeks, predominantly located at the sternum and left costal margin. The athlete did not report respiratory infections or unusual physical activities. He attributed his symptoms to musculoskeletal causes; consequently, physiotherapeutic treatment was commenced, with temporary subjective relief. However, while initially confined to exertion, the symptoms later progressed to occur at rest. On the night prior to presentation, the athlete experienced a single episode of vomiting but reported no other systemic symptoms such as fever or fatigue.

On physical examination, breath sounds were diminished at the left lung base, although evaluation was limited due to pain-related reduced respiratory excursion. Otherwise, findings were unremarkable with normal cardiac auscultation, heart rate (80/min), blood pressure (110/70 mmHg) and electrocardiogram (previously known first-degree atrioventricular block and inferolateral early repolarization pattern). Laboratory testing demonstrated marked systemic inflammation with a C-reactive protein level of 237 mg/L and leukocyte count of 11.19/nl, hypochromic microcytic anaemia (haemoglobin 10.8 g/dL), and mildly elevated high-sensitivity troponin T (21 pg/mL). Transthoracic ultrasound identified a fibrinous left-sided pleural effusion of 5 cm (*Figure 1*) and a mild circumferential pericardial effusion (8 mm end-diastolic posterior to the right ventricle) (*Figure 2*). Chest X-ray showed dystelectatic changes adjacent to the pleural effusion. Cardiac magnetic resonance imaging (CMRI) confirmed the diagnosis of acute pericarditis (*Figure 3*), with circumferential pericardial effusion (maximum extent of 9 mm end-diastolic), pericardial oedema (T2-weighted sequences) and late gadolinium enhancement (LGE). Anti-inflammatory therapy with ibuprofen 600 mg 1-1-1 and colchicine 0.5 mg 1-0-1 was initiated; due to borderline left ventricular ejection fraction (LVEF 52%) with high-normal ventricular end-diastolic diameters (left 56 mm, right 52 mm), mildly elevated *n*-terminal pro-B-type natriuretic peptide (262 ng/l) and questionable myocardial LGE (possibly respiratory artefact-related), guideline-directed heart failure therapy was commenced with bisoprolol 1.25 mg 1-0-1, candesartan 4 mg 1-0-1, and empagliflozin 10 mg 1-0-0.

**Figure 1:**
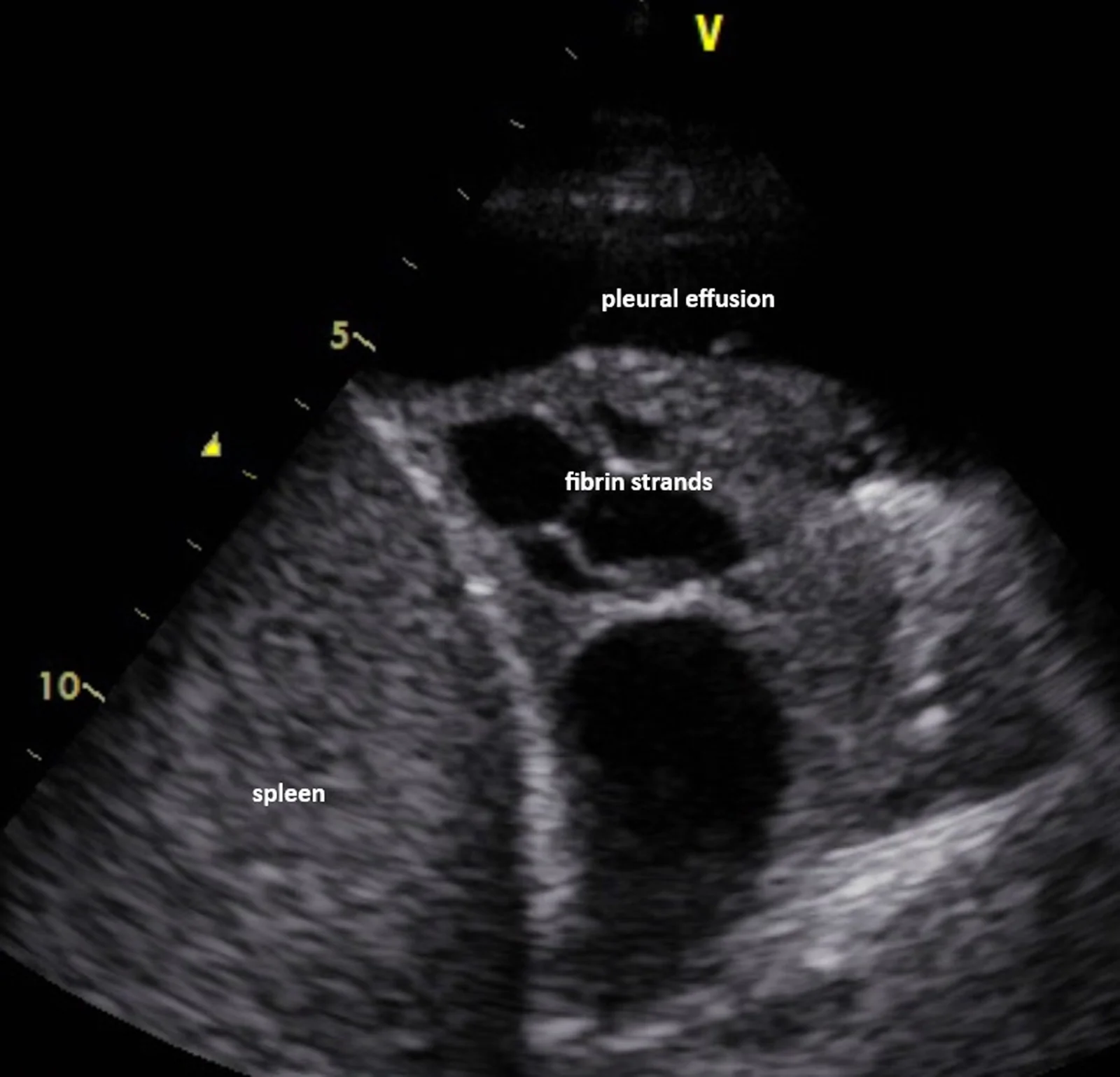
Sonographic image of the left pleural space with a pleural effusion of 5 cm with fibrinous strands.

**Figure 2:**
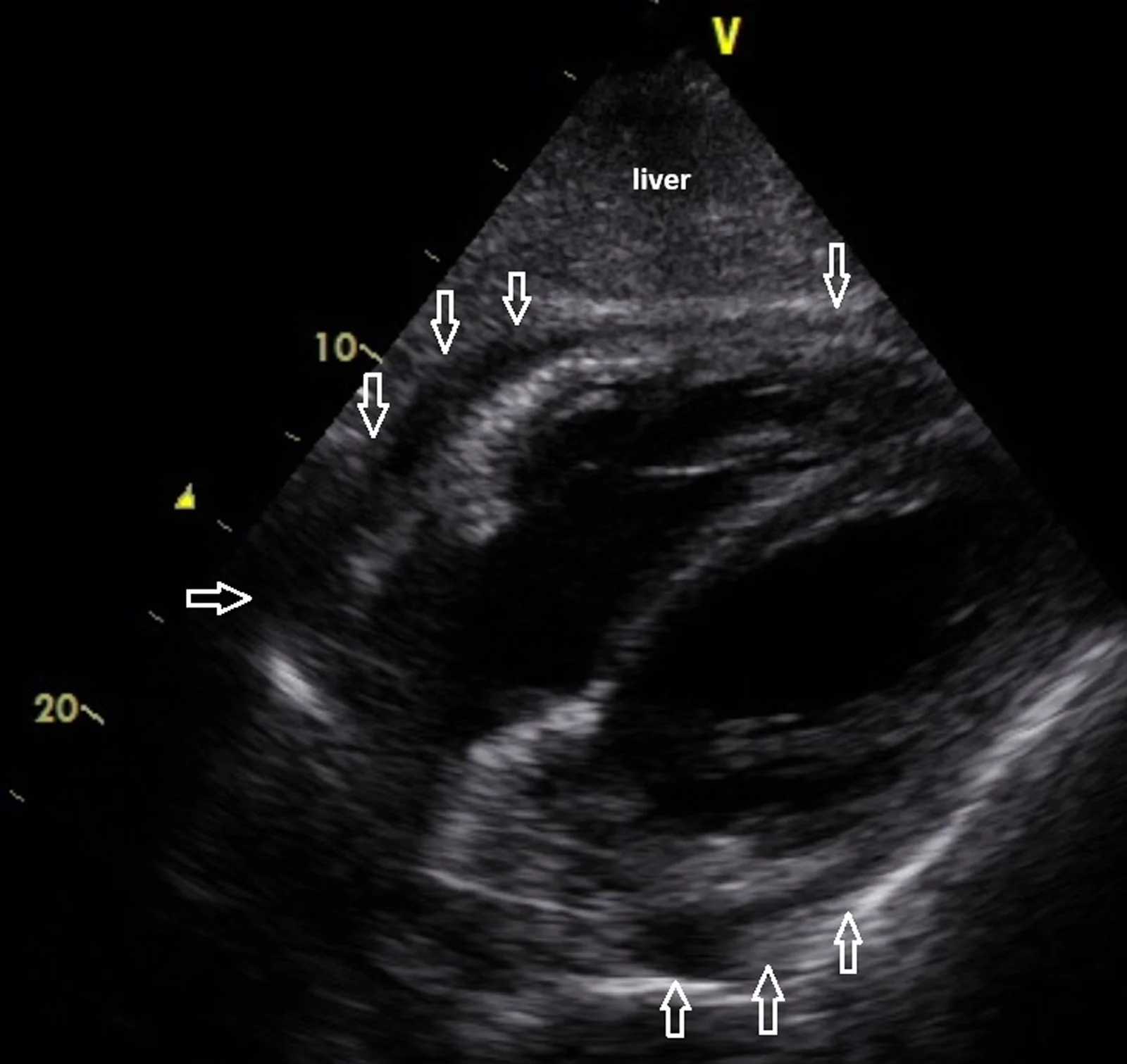
Sonographic image (subcostal view) of the heart with a mild circumferential pericardial effusion (white arrows).

**Figure 3:**
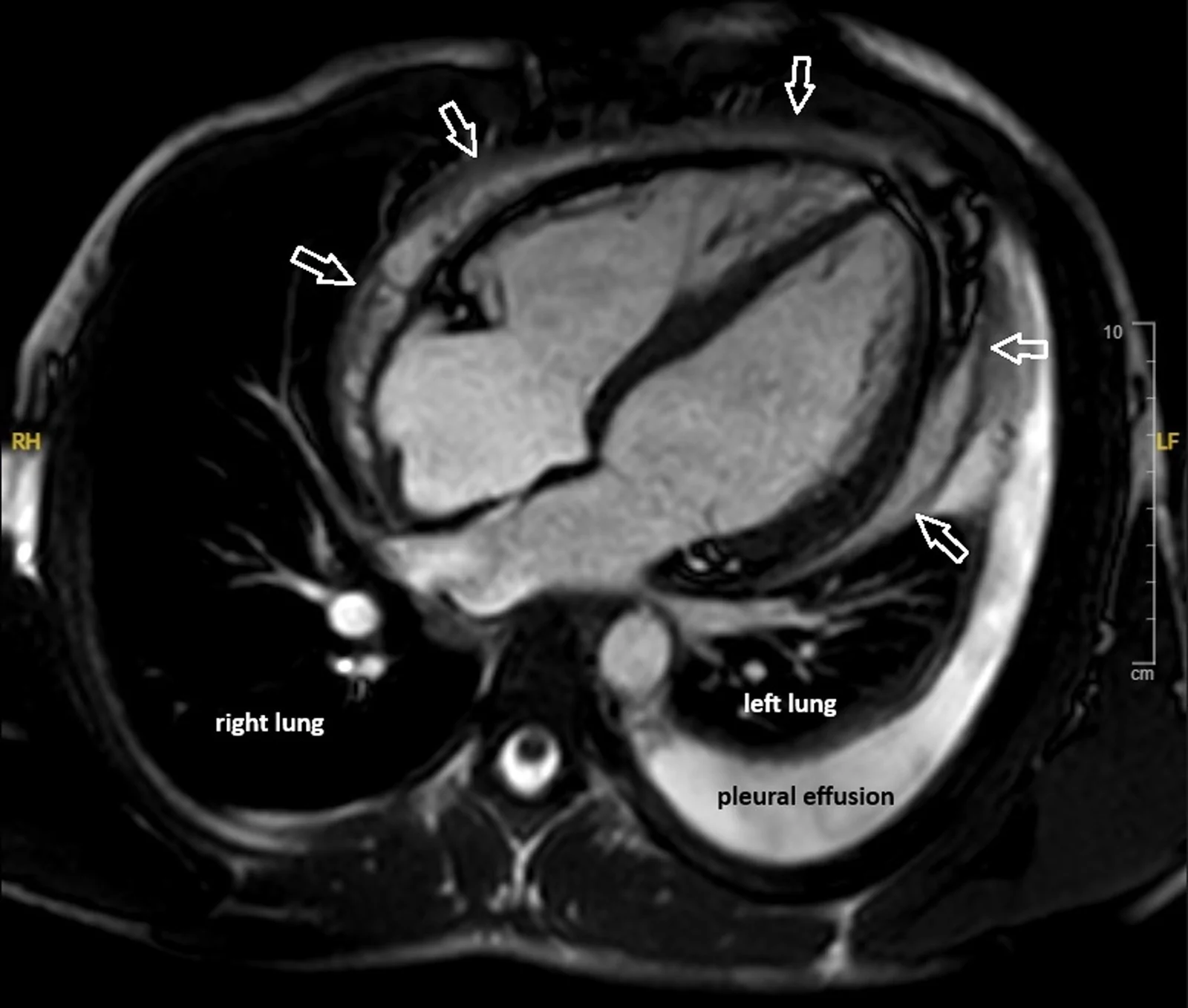
Cardiac magnetic resonance image showing a circumferential pericardial effusion (white arrows) as well as a pleural effusion with a collapsed left lung.

As no infectious origin was identified during the 5-day hospital stay (negative blood cultures, procalcitonin 0.09 ng/ml, unremarkable viral diagnostics (SARS-CoV-2, influenza A/B and respiratory syncytial virus), outpatient rheumatologic assessment was initiated. A highly elevated titre for anti-nuclear antibodies (ANAs 1:80.000) with a speckled pattern and specificity for ribonuclear proteins (anti-RNP antibodies) and mild Raynaud syndrome was suggestive for an autoimmune polyserositis secondary to mixed connective tissue disease. A 15-day corticosteroid-pulse-therapy (starting with 30 mg for 3 days followed by a tapering regimen) was additionally advised.

Follow-up assessments demonstrated clinical improvement and gradual normalization of sonographic imaging and laboratory findings. Pericardial effusion and anaemia were no longer detectable after 10 days, while pleural effusion had resolved within 3 weeks. A normal CRP level was first noted after 6 weeks. Ibuprofen therapy was discontinued entirely at this point.

After 3 months of sports restriction, follow-up CMRI demonstrated normal LVEF (58%) with no evidence of active inflammation but showed significant circumferential pericardial LGE (*Figure 4*). As no myocardial LGE was detectable here, a gradual return-to-sport protocol was implemented (starting with low-intensity strength training (progressing to submaximal) and aerobic endurance training (predominantly on cycle ergometer or swimming). Colchicine was reduced to a once-daily dose; heart failure therapy was gradually tapered (first bisoprolol was withdrawn, then empagliflozin). A first maximal exercise test was performed after 4 months, and since the findings were unremarkable, the athlete was then medically cleared for intensive exercise (*e.g*. maximal strength training, interval endurance training). With persistently normal CRP and absence of clinical symptoms, colchicine and candesartan were discontinued after 6 months.

**Figure 4:**
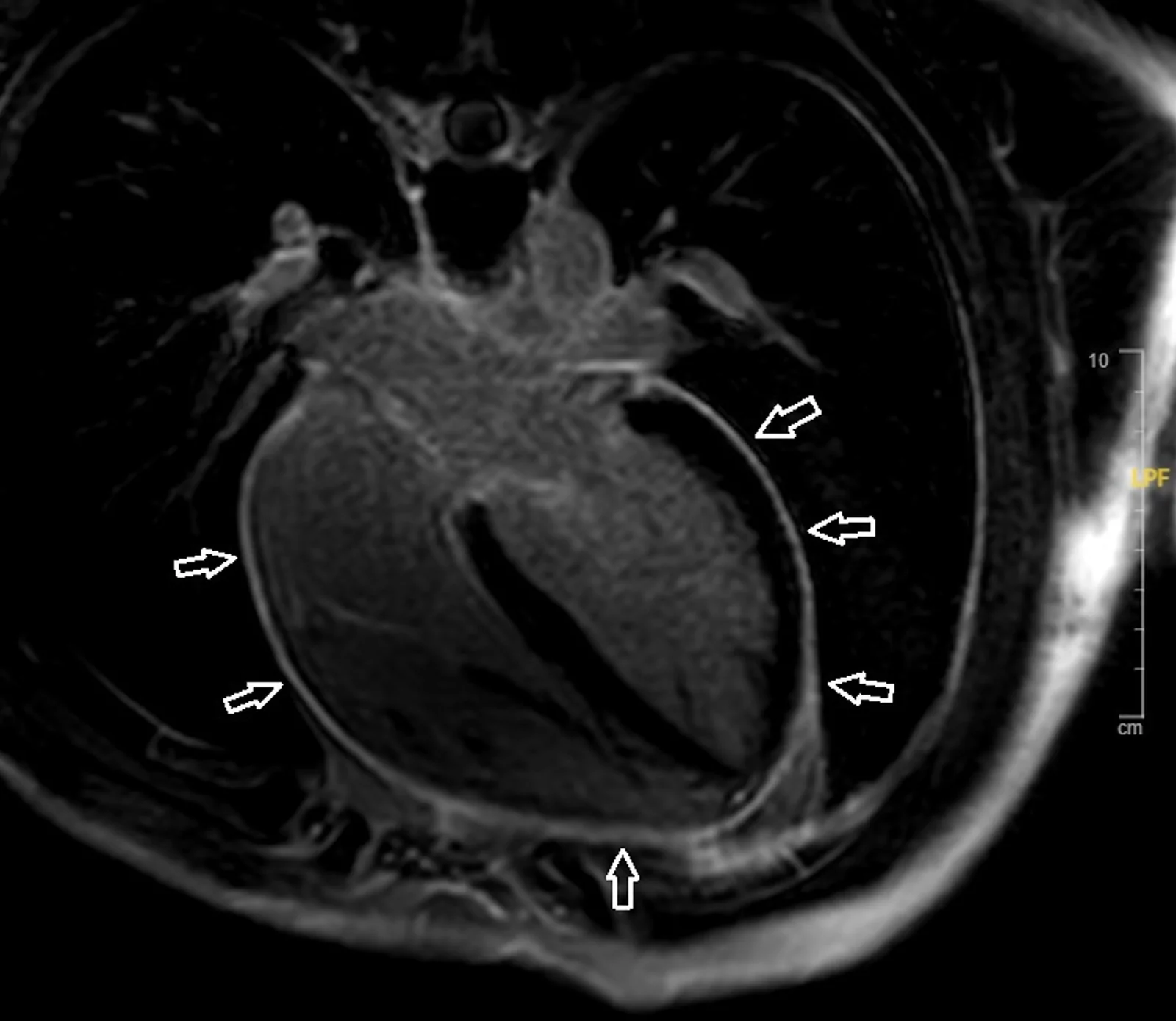
Cardiac magnetic resonance image showing significant pericardial late gadolinium enhancement (white arrows).

The athlete has since remained clinically stable and reached his previous performance capacity after 7 months (VO_2_max 46.3 mL/kg/min). The first competitive appearance took place 8 months after diagnosis.

## Discussion

Acute pericarditis accounts for up to 5% of emergency department visits for non-ischaemic chest pain in North America and Western Europe.^2^ A rheumatologic aetiology of acute pericarditis is present in 2–7% of these patients.^3^ The overall incidence rate of acute pericarditis increases with age (median age in clinical series of 56 years).^4^

Thus, the likelihood that a young athlete presenting with chest pain is diagnosed with autoinflammatory pericarditis is very low. In addition, the high tolerance of the expected symptoms given the markedly elevated inflammatory parameters is notable, as the athlete was still able to compete in a professional-level rugby match just a few days prior to presentation. Therefore, this case highlights the importance of thorough diagnostic evaluation of chest pain in young athletes.

The ESC guidelines on sports cardiology recommend that sports after acute pericarditis can be resumed once the patient is free of symptoms and the active inflammation has completely resolved, depending on severity after 30 days to 3 months.^5^ In our case, inflammatory markers returned to the normal range after 6 weeks and the athlete was also free of symptoms. However, due to the significant findings with initially uncertain myocardial involvement, we decided to perform a follow-up MRI after 3 months and only then begin the return-to-sport protocol.

Given the borderline LVEF, guideline-directed low-dose heart failure therapy was initiated besides anti-inflammatory treatment. A low-normal to mildly reduced LVEF in the presence of high-normal ventricular dimensions may also be observed in the context of an athlete’s heart,^6^ which represents cardiac adaptation to intense physical activity. Therefore, the necessity of heart failure therapy in this case could at least be debated. However, given the good tolerability and the temporary restriction from sports participation, the potential benefits prevailed.

Another factor prompting a somewhat more cautious approach was the reportedly high recurrence rate of pericarditis in young adults with an underlying autoimmune aetiology.

After a first episode of idiopathic or viral pericarditis, recurrence may occur in 15–30% of patients, mostly within the first 3–6 months.^7^ An underlying autoimmune disease is even associated with an approximately 50% increased risk of recurrent pericarditis.^8^ Also, a high pericardial LGE burden—as seen in the follow-up MRI—is likewise associated with an increased recurrence rate.^9^

This raises the question of how to manage the inherently high recurrence rate in the context of competitive sports. We decided to explicitly inform the athlete about the high risk of recurrence and agreed on follow-up monitoring of inflammatory markers every 4–6 weeks (earlier if any symptoms recur) to detect a recurrence at least at an early stage. Rheumatologic follow-up was performed every 4 months. To date, the athlete has experienced no recurrence.

The influence of high physical activity on the potential risk of developing a constrictive pericarditis remains also uncertain. In autoimmune cases the risk is reported to be 2–5%.^2^ However, data on athletes are missing here.

In summary, rare entities such as autoimmune pericarditis should be included in the differential diagnosis of chest pain, even in young athletes. In the context of competitive sports the high recurrence risk requires close monitoring during and after the return-to-sport protocol and justifies a somewhat more cautious approach.

## Lead author biography


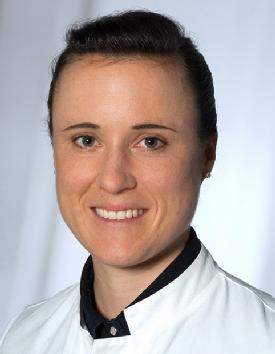
Dr. med. Theresa Maria Betz is a senior physician at the Department of Sports Medicine, Heidelberg University Hospital, Germany. She is board-certified in internal medicine and cardiology with additional certification in sports medicine. Alongside her clinical work, she serves as team physician for the TSG Hoffenheim women’s football team. Her clinical and research interests focus on sports cardiology, exercise physiology, cardiovascular prevention, and athlete health.

## Data Availability

Data are available upon reasonable request from the corresponding author.
